# Repurposing levocetirizine hydrochloride loaded into cationic ceramide/phospholipid composite (CCPCs) for management of alopecia: central composite design optimization, *in- silico* and *in-vivo* studies

**DOI:** 10.1080/10717544.2022.2108939

**Published:** 2022-09-01

**Authors:** Rofida Albash, Rania Moataz El-Dahmy, Mohammed I. A. Hamed, Khaled M. Darwish, Abdulrahman M. Alahdal, Amira B. Kassem, Abdurrahman M. Fahmy

**Affiliations:** a Department of Pharmaceutics, College of Pharmaceutical Sciences and Drug Manufacturing, Misr University for Science and Technology, 6th of October City, Egypt; b Department of Pharmaceutics and Industrial Pharmacy, Faculty of Pharmacy, October 6 University, Cairo, Egypt; c Department of Organic and Medicinal Chemistry, Faculty of Pharmacy, Fayoum University, Faiyum, Egypt; d Department of Medicinal Chemistry, Faculty of Pharmacy, Suez Canal University, Ismailia, Egypt; e Department of Pharmacy Practice, Faculty of Pharmacy, King Abdulaziz University, Jeddah, Saudi Arabia; f Department of Clinical Pharmacy and Pharmacy Practice Faculty of Pharmacy, Damanhour University, Damanhour, Egypt; g Department of Pharmaceutics and Industrial Pharmacy, Faculty of Pharmacy, Cairo University, Giza, Egypt

**Keywords:** Alopecia, levocetirizine hydrochloride, *in-silico* study, ceramide, drug discovery, industrial development

## Abstract

Levocetirizine hydrochloride (LVC) is an antihistaminic drug that is repurposed for the treatment of alopecia. This investigation is targeted for formulating LVC into cationic ceramide/phospholipid composite (CCPCs) for the management of alopecia. CCPCs were fabricated by ethanol-injection approach, through a central composite experiment. CCPCs were evaluated by inspecting their entrapment efficiency (EE%), polydispersity index (PDI), particle size (PS), and zeta potential (ZP). The optimum CCPCs were additionally studied by *in-vitro*, *ex-vivo*, *in-silico*, and *in-vivo* studies. The fabricated CCPCs had acceptable EE%, PS, PDI, and ZP values. The statistical optimization elected optimum CCPCs composed of 5 mg hyaluronic acid, 10 mg ceramide III, and 5 mg dimethyldidodecylammonium bromide employing phytantriol as a permeation enhancer. The optimum CCPCs had EE%, PS, PDI, and ZP of 88.36 ± 0.34%, 479.00 ± 50.34 nm, 0.377 ± 0.0035, and 20.20 ± 1.13 mV, respectively. The optimum CCPC maintained its stability for up to 90 days. It also viewed vesicles of tube shape via transmission electron microscope. The *in-silico* assessment resulted in better interaction and stability between LVC and vesicle components in water. The *ex-vivo* and *in-vivo* assessments showed satisfactory skin retention of LVC from optimum CCPCs. The histopathological assessment verified the safety of optimum CCPCs to be topically applied. Overall, the optimum CCPCs could be utilized as a potential system for the topical management of alopecia, with a prolonged period of activity, coupled with reduced LVC shortcomings.

## Introduction

Alopecia is a state, that produces hair loss from the head or various body parts, as hair is presumed to exist naturally. Alopecia areata (AA), androgenic alopecia (AGA), and chemotherapy-induced alopecia are three of the most frequent types of alopecia (Rambwawasvika et al., 2021). T cells directed to hair follicles are thought to be involved in AA, which is regarded as an autoimmune disease (Madani & Shapiro, 2000). It is worth mentioning that AGA associated with males is an androgen-related disorder, and a hereditary predisposition is found, however, androgens’ role in female AGA is still unknown (Finner, 2011).

It was previously presumed that prostaglandin (PGs) pathways have an impact on AGA etiology (Arias-Santiago et al., 2012). Cyclooxygenase (COX) causes PGs to transform from arachidonic acid to unstable prostaglandin H_2_, which is subsequently produced by the respective prostaglandin synthetases into a variety of active prostaglandins (e.g. PGD_2_, PGF_2_a, PGE_2_, PGI_2_, and prostacyclin) or thromboxane A_2_ (TXA_2_). PGE_2_ and PGF_2_ can enhance hair development, on the other hand, PGD_2_ can prohibit hair growth and increase hair follicle shrinkage, according to studies comparing the impacts of various PG subtypes on hair growth. In addition, PGD_2_ also activates androgen receptors by modulating the DP_2_ and AKT/GSK_3_ signaling pathways in human dermal papilla cells (Jeong et al., 2018).

Although hormone regulators, vasodilators, and immunomodulators are among the medications used to treat alopecia, few treatments can effectively increase hair growth. Finasteride and minoxidil are still the most widely prescribed medicines for alopecia. Unfortunately, finasteride has been linked to sexual adverse effects, while minoxidil has been linked to irritating dermatitis, headaches, and hypotension (Wen et al., 2020).

Levocetirizine hydrochloride (LVC) is a histamine H_1_ receptor antagonist that has been employed to treat cutaneous, respiratory, and ocular allergies. It binds to transmembrane G protein-coupled receptors, inhibiting the release of inflammatory mediators linked to allergies. LVC, as the R-enantiomer of cetirizine, has greater bioavailability, as well as a plasma binding rate of up to 95%. It has a low tissue affinity, is cardiotoxic, and has a modest sedative impact (Kanei et al., 2014). A recent study by Rossi et al., 2018 found that applying 1% cetirizine to the balding area enhanced hair density. Another study on human dermal papilla cells found that LVC inhibited the PGD_2_-GPR_44_ path while activating the AKT signaling path, promoting hair growth and proliferation *in-vitro* (Dena & Gaber, 2017). Moreover, a previous investigation done by wen et al. inspected the impacts of LVC on hair proliferation, and confirmed the ability of LVC to maintain hair growth (Wen et al., 2020). LVC was previously reported to be formulated into nanovesicles (transethosomes) for topical treatment of atopic dermatitis to overcome drug shortcomings such as drowsiness, tiredness, dry mouth, fever, and cough (Goindi et al., 2014). On the other hand, the topical route provided a suitable solution to overcome LVC’s unacceptable taste problem (Amelian et al., 2017).

Hyaluronic acid-enriched cerosomes (HAECs) are regarded as a new type of vesicle that has shown great success in topical drug delivery. A previous study confirmed the potency of topical delivery of spironolactone using HAECs (Albash et al., 2021a). Both hyaluronic acid (HA) and ceramide are added to nanovesicles to augment the skin deposition of drugs (Albash et al., 2021a; 2021b). In a view of this, we represent the preparation, and characterization of cationic ceramide/phospholipid composite (CCPCs) for the management of alopecia. In addition, phytantriol is utilized in this investigation to augment skin penetration of LVC. Phytantriol is a lipid commonly employed in cosmetic manufacturing for hair and skin maintenance and is thought to improve skin penetration (Bender et al., 2005). Moreover, Dimethyldidodecylammonium bromide (DDAB) being a cationic surfactant was utilized to fabricate CCPCs that might be able to enhance skin deposition and retention of the prepared vesicles as previously reported in the literature (Oh et al., 2011). The electrostatic interaction through the negative epidermis layer and the cationic vesicles, which is thought to promote transdermal absorption, was possibly responsible for the retention in deep epidermal regions (Lin et al., 2018).

To the best of our knowledge, there is no scientific paper discussing the role of CCPCs to augment the deposition of LVC for the management of alopecia. As a result, this investigation intended to assess CCPCs’ capability to improve LVC topical retention and analyze its safety. To achieve this, numerous variables impacting vesicle features were investigated using central composite design (CCD) design by Design Expert® software to select the optimum CCPCs. CCD, as well recognized as response surface methodology, is a quick method for determining the relationship between an experimental response and a collection of input variables. It might also establish the optimal level of experimental elements necessary for a specific reaction. A factor is a variable that can have its value changed throughout an experiment. The response variable is a numerical value that is influenced by the factor levels. The number of runs mandatory to establish a mathematical statistics in the experimental design region is reduced with CCD (Sun & Zhang, 2004). Ceramide amount (X_1_), HA amount (X_2_), and DDAB amount (X_3_) were studied as independent variables, while entrapment efficiency percentage (EE%; Y_1_), particle size (PS; Y_2_), and zeta potential (ZP; Y_3_), were selected as dependent variables. The optimum CCPCs were assessed for their shape and stability and related to LVC solution in *ex-vivo* permeation study. *In-silico* study was performed for the optimum formula components to investigate their stability during their binding. Further, histopathological, and dermatokinetic studies of LVC released from the optimum CCPCs compared to LVC solution were performed in male Wistar rats.

## Materials

Levocetirizine (LVC) was gifted from Global Napi Pharmaceutical Co. (Cairo, Egypt). L-α phosphatidylcholine (PC) and dimethyldidodecylammonium bromide (DDAB) were purchased from Sigma-Aldrich (St. Loius, MO, U.S.A.). Hyaluronic acid (HA) was purchased from Acros Organics, Belgium. Ceramide III was supplied by Evonik Co. (Germany). Phytantriol was obtained as a gift from DSM (Basel, Switzerland). Ethanol and methanol HPLC grade were obtained from Merck (Darmstadt, Germany).

## Methods

### Preparation of LVC loaded CCPCs

CCPCs were fabricated using a modified ethanol injection method. PC, ceramide, phytantriol, and DDAB were liquified in 4 mL of a 1:1 ethanol/chloroform mix (Albash et al., 2021a). The organic mixture was added to a heated (60 °C) distilled water (10 mL) containing both LVC (25 mg) and HA (25 mg). The produced mixture was agitated for 30 minutes at 1500 rpm on a magnetic stirrer (Model MSH-20D, GmbH, Germany), then the formulations were stored in the refrigerator.

### Characterization of LVC loaded CCPCs

#### Determination of entrapment efficiency percentage (EE%)

Employing a cooling centrifuge (Sigma 3 K 30, Germany), the CCPCs dispersion for the prepared formulae was centrifuged at 20,000 rpm for 1 hour at 4 °C. Then, the sediment was lysed using methanol and analyzed at λ_max_ 231 nm using a UV-Vis spectrophotometer (Shimadzu UV1650 Spectrophotometer, Kyoto, Japan). The following equation was used to get the EE%:( Abdellatif et al., 2017; El-dahmy et al., 2021)

EE% = $\left(\frac{\text{Entrapped LVC concentration}}{\text{Total LVC concentration}}\right)$ x 100 (1)

#### Determination of particle size (PS), polydispersity index (PDI), and zeta potential (ZP)

Using a Malvern Zetasizer 2000 (Malvern Instruments Ltd., Malvern, UK), the PS and PDI of vesicle dispersions were assessed for the produced formulations (Albash et al., 2021c). After dilution, the readings were taken. The electrophoretic movement of the particles was studied as part of the ZP measurement. All measurements were made three times to ensure accuracy (Imam et al., 2015; Elshafeey & El-dahmy, 2021; Albash et al., 2022).

#### Optimization design

CCD was created to assess the impact of a variety of parameters on the formulation of CCPCs using Design Expert^®^ (Stat Ease, Inc., Minneapolis, MN, USA). A total of 20 runs were required by the design. Three factors were studied: Ceramide amount (X_1_), HA amount (X_2_), and DDAB amount (X_3_) were opted as independent variables, as EE% (Y_1_), PS (Y_2_), and ZP (Y_3_) were chosen as dependent variables (Table 1). Afterward, the opted formula selection relied on the desirability function, which allowed the investigation of all responses at the same time. The election was decided to obtain a suggestion with the least PS and the highest EE% and ZP. The solution with the highest desirability (almost one) was chosen (Table 1). The selected formula was further characterized.

**Table 1.** Central composite design used for optimization of LVC-loaded CCPCs.

Factors (Independent variables)	Factor type	Levels		
		**(**−**1)**		(+1)
X_1_: Ceramide amount (mg)	Numeric	10		30
X_2_: HA (mg)	Numeric	5		15
X_3_: DDAB amount (mg)	Numeric	5		15
Responses (Dependent variables)		Desirability Constraints		
Y_1_: EE%		Maximize		
Y_2_: PS (nm)		Minimize		
Y_3_: ZP (mV)		Maximize (absolute value)		

Abbreviation: HA: hyaluronic acid; EE%: entrapment efficiency percent; DDAB: dimethyldidodecylammonium bromide; LVC: levocetirizine hydrochloride; PS: particle size; ZP: zeta potential, and CCPCs; cationic ceramide/phospholipid composite.

#### Stability study

The optimum CCPCs’ physical stability was explored to track the degree of vesicle growth formation, drug leakage, and any other physical alternations. The optimum CCPCs were preserved at 4 °C for 3 months, and their stability was assessed by associating the PS, PDI, EE%, and ZP of the stored formula with the freshly fabricated formula. The system was also examined for any particle sedimentation (Albash et al., 2021b). Statistical significance was analyzed by Student’s t-test using SPSS^®^ software 22.0. Difference at *P* ≤ 0.05 was considered significant.

### Transmission electron microscopy (TEM)

TEM (Joel JEM 1230, Tokyo, Japan) was used to investigate the morphology of the best CCPCs. The samples were deposited on a carbon-coated copper grid as a film, dyed with phosphotungstic acid 1.5%, and then observed (El-Dahmy et al., 2014; Imam et al., 2017; Albash et al., 2021c).

### *In-silico* studies

The docking protocol proceeded through a validated comprehensive workflow described within our previous study (Albash et al., 2021a). Briefly, the isomeric molecular structure of LVC and the optimum CCPCs additives; PC, ceramide III, phytantriol DDAB, and HA were constructed and energy minimized (gradient RMS 1 × 10^−5^ Kcal/mol.A^2^ at Amber10:EHT forcefield) via the MOE software (CCG, Montreal, Canada). Docking of the drug as well as the optimum CCPCs additives sequentially proceeded on the PC target molecule through the triangular-matcher approach and London/dG ranking scoring system. The ten top-scored binding modes were subsequentially refined via energy minimization within the target interface before being restored using the Generalized Born-solvation_VI/Weighted-Surface Area/dG forcefield. The latter scoring relied on van der Waals hydrophobic, Coulomb’s electrostatic potentials, electrostatic solvation potentials, loaded partial charges, and exposure-weighted surface area (Vilar et al., 2008). The predicted ligand/target complex was selected based on favored docking energies (high negative-valued Kcal/mol) in addition to obtaining relevant/strong intermolecular binding contacts (polar/hydrophobic) between the investigated molecules. Optimum hydrogen bonding was set at 3.0 Å bond length and 20° bond angle thresholds, while ≤ 5 Å was assigned for the hydrophobic contacts (de Souza et al., 2019; Albuquerque et al., 2020).

Explicit molecular dynamics simulation proceeded for the predicted ligand-target complex through MOE software under the explicit Amber 10: EHT forcefield. The system was solvated in a water cubic box (45 × 45 × 45 Å; 3329 water molecules). The solvated system (Table 2) was minimized for 0.5 ns under a constant number of particles, Volume, and Temperature; 310 K (NVT) ensemble, then equilibrated for another 0.5 ns under a constant number of particles, Pressure 101 kPa, and Temperature 310 K (NPT) ensemble. Finally, the system was produced for explicit 1 ns molecular dynamics simulation under the NPT ensemble. Analysis of the MD trajectories was proceeded through the MOE Database Calculator for estimating the average interaction potential energy between LVC and PC. Snapshots at regular 0.2 ns-intervals were extracted and graphically represented using Schrödinger-PyMol software for evaluating the time-evolution conformational changes of the ligand-target complex along with the simulated timeframe.

**Table 2.** The atomic composition of the molecular dynamics simulated LVC-loaded CCPCs.

Solvation State	Atomic composition (atom numbers)							
	LVC	PC	Ceramide III	Phytantriol	DDAB	HA	Water	Total
**100% Water**	1 × 52	1 × 134	1 × 112	1 × 65	1 × 83	1 × 49	3 × 3359	10,572

Abbreviation: HA: hyaluronic acid; DDAB: dimethyldidodecylammonium bromide; LVC: levocetirizine hydrochloride, PC; phospholipid, and CCPCs; cationic ceramide/phospholipid composite.

### *Ex-vivo* studies

The permeation of LVC, through new-born rat skin, from CCPCs, related to LVC solution was performed. The skin was attached to a plastic dialysis tube with a 3.14 cm^2^ permeation area. 1 ml from CCPCs equivalent to 2.5 mg LVC was supplied into the donor chamber while the permeation media was 50 ml of phosphate buffer (pH= 5.5) at 37 ± 0.5 °C stirred at 100 rpm. 1 mL samples were taken at 1, 2, 4, 6, 8, and 24 hours and then analyzed using HPLC (Arayne et al., 2008). Per unit area, the total amount penetrated was computed. Afterwards, the maximum flux values at 24 h (*J*_max_) were gained (El-dahmy et al., 2021). The skin was cleaned and separated before being vortexed with 5 mL methanol. After one cycle of sonication for 90 minutes, the skin was centrifuged at 10000 rpm for 15 minutes, and the LVC concentration was analyzed to quantify the amount deposited after 24 hours (Dep_24_). Finally, the ratio of LVC deposited within the skin to that transported via the skin was calculated as the local accumulation efficiency index (LAEI) for LVC (Albash et al., 2021a). Statistical significance was analyzed by Student’s t-test using SPSS^®^ software 22.0. Difference at *P* ≤ 0.05 was considered significant.

### *In-vivo* studies

#### Animals

The study protocol was approved by Research Ethics Committee (REC) for experimental and clinical studies at Faculty of Pharmacy, Cairo University (reference number = (PI) 3045). The use and handling of animals in all studies complied with the EU directive 2010/63/EU for animal experiment. Male Wistar rats (150–200 gm), with an average age of 7 weeks were utilized. Bottle caps having an area of 4.91 cm^2^ were used as drug pools for the delivery of LVC solution and CCPCs in *in-vivo* investigations. The bottle tops were attached to previously shaved dorsal rat skin (Albash et al., 2019).

#### In-vivo dermatokinetic assessment

The animals were placed into two groups, each with 18 animals. Group I was given LVC solution, while group II was given the optimum CCPCs topically. 1 mL from LVC solution and the optimum CCPCs equivalent to 2.5 mg LVC were applied to rat skin (Goindi et al., 2014). Three animals from each group were slaughtered at varied time intervals after treatment (1, 2, 4, 6, 8, and 10 hrs). The animal carcasses were burned after the removal of their skin. The removed skin was cut into fragments and sonicated for 30 minutes in 5 mL methanol. HPLC was used to determine the concentration of LVC after the extract was filtered through a 0.45 filter (Arayne et al., 2008). Dermatokinetic data was investigated and dermatokinetic parameters such as T_max_, C_max,_ and AUC_0–10_ were premeditated operating Kinetica^®^ software (Albash et al., 2021b). C_max_ and AUC_0–10_ for the previously treated groups were compared with student’s-t-test using SPSS^®^ software 22.0, a nonparametric signed-rank test (Mann-test) Whitney’s was utilized to relate the medians of T_max_ for the treated groups.

#### Histopathologic evaluation

A total of nine rats were divided into three groups, each with three rats, and the treatment lasted one day. Group I served as a control, whereas groups II and III were given LVC solution, and the optimum CCPCs, respectively. Samples were autopsied and fixed in 10% formalin for one day before being washed and dehydrated. Specimens were cleaned with xylene, preserved in paraffin blocks, and sectioned at 4 mm using a microtome (Leica Microsystems SM2400, Cambridge, UK). Light microscopy was used to view the specimens after they had been deparaffinized, stained, and histopathologically inspected (Axiostar plus, ZEISS, Oberkochen, Germany) (Albash et al., 2019).

## Results and discussion

### Analysis of central composite design

The developed formulae were optimized using CCD employing Design-expert^®^. To develop CCPCs, the software generated 20 experimental runs (Table 3). The model chosen was quadratic for EE%, PS, and ZP. Adequate precision is used to affirm that the model might be used to navigate the design space. A ratio superior to four is preferred which was noticed for all dependent variables (Table 4). The predicted R^2^ values were in a good harmony with the adjusted R^2^ in all dependent variables.

**Table 3.** Experimental runs, independent variables, and measured responses of LVC-loaded CCPCs.

F	Ceramide amount (mg)	HA amount (mg)	DDAB amount (mg)	EE%	PS (nm)	PDI	ZP (mV)
**F1**	10	5	5	88.36 ± 0.34	479.00 ± 50.34	0.377 ± 0.003	20.20 ± 1.13
**F2**	10	5	15	62.57 ± 0.71	342.00 ± 0.21	0.292 ± 0.061	26.40 ± 0.49
**F3**	10	10	10	75.51 ± 6.05	438.00 ± 13.08	0.493 ± 0.013	13.20 ± 1.13
**F4**	10	15	5	84.23 ± 4.86	400.00 ± 28.28	0.262 ± 0.004	15.80 ± 0.28
**F5**	10	15	15	69.63 ± 1.46	603.00 ± 53.74	0.530 ± 0.060	19.40 ± 0.35
**F6**	20	5	10	68.00 ± 4.00	756.80 ± 40.16	0.182 ± 0.0021	27.60 ± 0.91
**F7**	20	10	5	76.96 ± 5.16	592.45 ± 37.08	0.179 ± 0.036	10.00 ± 0.41
**F8**	20	10	10	61.00 ± 1.00	700.60 ± 7.84	0.271 ± 0.016	14.70 ± 1.97
**F9**	20	10	10	60.50 ± 0.45	720.60 ± 56.49	0.165 ± 0.024	17.50 ± 0.42
**F10**	20	10	10	69.61 ± 0.34	679.60 ± 20.93	0.270 ± 0.090	10.50 ± 0.84
**F11**	20	10	10	64.29 ± 2.19	700.60 ± 29.76	0.160 ± 0.028	12.50 ± 0.56
**F12**	20	10	10	69.62 ± 0.24	720.60 ± 14.14	0.260 ± 0.007	15.50 ± 0.28
**F13**	20	10	10	67.84 ± 0.54	679.60 ± 20.93	0.150 ± 0.010	16.90 ± 0.14
**F14**	20	10	15	60.35 ± 0.63	572.90 ± 12.02	0.591 ± 0.026	15.70 ± 0.49
**F15**	20	15	10	70.11 ± 1.43	740.70 ± 27.71	0.342 ± 0.054	16.70 ± 0.42
**F16**	30	5	5	80.52 ± 3.08	853.20 ± 9.33	0.325 ± 0.036	20.70 ± 0.77
**F17**	30	5	15	70.60 ± 3.95	536.40 ± 19.79	0.336 ± 0.030	34.40 ± 1.55
**F18**	30	10	10	82.55 ± 3.67	687.50 ± 26.51	0.141 ± 0.060	19.40 ± 0.28
**F19**	30	15	5	86.53 ± 5.16	745.80 ± 14.14	0.251 ± 0.009	7.56 ± 0.48
**F20**	30	15	15	89.32 ± 3.50	708.30 ± 12.94	0.556 ± 0.080	20.80 ± 0.07

Presented values are the mean ± SD (*n* = 3).

^a^All formulae contained 25 mg LVC and 100 mg phosphatidylcholine, in a volume of 10 ml.

Abbreviation: HA: hyaluronic acid; DDAB: dimethyldidodecylammonium bromide; EE%: entrapment efficiency percent; PS: particle size; PDI: polydispersity index; ZP: zeta potential; LVC: levocetirizine hydrochloride, and CCPCs; cationic ceramide/phospholipid composite.

**Table 4.** Output data of the central composite design and predicted and observed values for the optimum CCPCs.

Responses	EE%	PS (nm)	ZP (mV)
**Adequate precision**	14.25	22.24	20.39
**Adjusted *R*^2^**	0.887	0.944	0.861
**Predicted *R^2^***	0.866	0.849	0.849
**Significant factors**	X_1_, X_2_, X_3_	X_1_, X_2_, X_3_	X_2_, X_3_
**Predicted value of the selected formula**	88.84	484.53	21.84
**Observed value of the selected formula**	88.36	479.00	20.20

Abbreviations: EE%, entrapment efficiency percentage; CCPCs, cationic ceramide/phospholipid composite; PS, particle size, and ZP; zeta potential.

### Effect of formulation variables on the EE%

**Figure 1.**
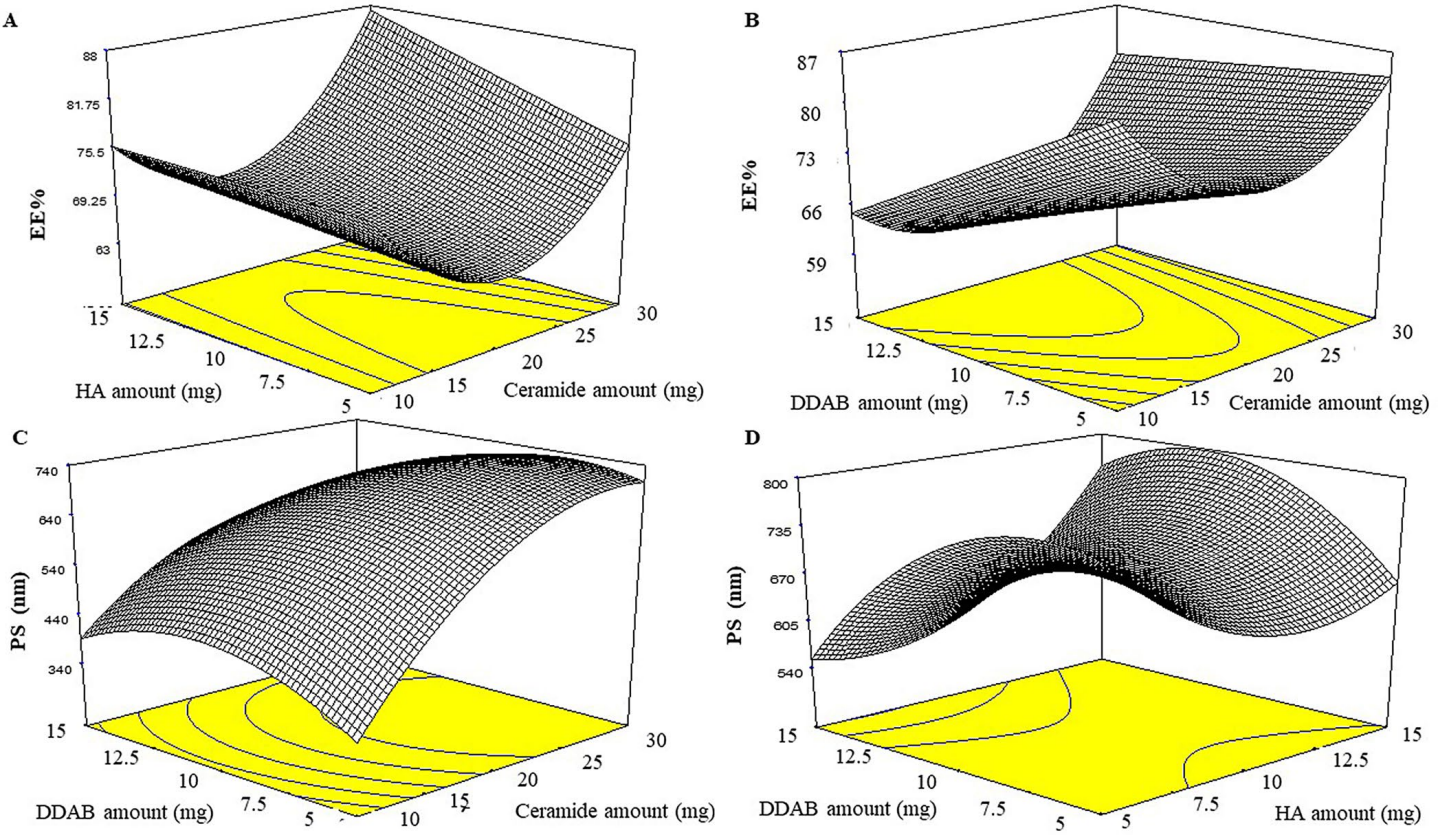
Effect of formulation variables on EE% of LVC-CCPCs (A-B) and PS (C-D). Abbreviation: EE%: Entrapment efficiency percentage, PS: Particle size, LVC: levocetirizine hydrochloride and CCPCs; cationic ceramide/phospholipid composite.

EE% of LVC-loaded CCPCs ranged from 60.35 ± 0.63 to 89.32 ± 3.50% (Table 3). The output data (Table 4), and Figure 1A–B shows the outcome of the premeditated variables on the EE% of the CCPCs. All factors, ceramide amount (X_1_), HA amount (X_2_), and DDAB amount (X_3_) significantly impacted the EE% of the fabricated formulae (*p* = 0.0102 for ceramide amount *p* = 0.012 for HA amount, and DDAB amount < 0.0001). Regarding ceramide amount (X_1_), EE% of CCPCs augmented by increasing ceramide amount as it has previously been noted that rising the amount of ceramide in the dispersion improves its viscosity. Hence, the drug diffusion to the external aqueous phase will be hindered by the highly viscous formulations, resulting in higher EE% values (Albash et al., 2021b). For HA amount (X_2_), it was found that EE% increased by using HA at high concentrations. The improvement in EE% could be attributed to HA’s hydrophilic domain, which could allow more hydrophilic drugs to be loaded effectively (Xie et al., 2018). Considering the DDAB amount (X_3_), it was found that by increasing the DDAB amount from 5 to 15 mg the amount of LVC entrapped decreased. The previous findings were in accordance with Albash et al., 2021d as they described upon increasing cationic SAA as DDAB the EE% of leciplex loaded Moxifloxacin hydrochloride decreased. In addition, they correlate the lower EE% values at high DDAB concentration owed to the enhancement of PC solubility by SAA, promoting the drug leak from vesicles. On the other hand, the higher EE% values at low DDAB concentrations could be ascribed to the formation of tight bilayers around LVC and the amount of SAA was not enough to solubilize PC bilayers.

### Effect of formulation variables on the PS

PS of LVC-loaded CCPCs fluctuated from 342.00 ± 0.21 to 853.20 ± 9.33 nm (Table 3). The output data (Table 4), and Figure 1C–D show the effect of the studied variables on the ZP of the CCPCs. All factors showed a significant effect on the response with p values of < 0.0001, 0.0411, and 0.0103 for ceramide amount (X_1_), HA amount (X_2_), and DDAD amount (X_3_), respectively. Considering ceramide amount (X_1_), the PS of the prepared CCPCs increased by increasing the ceramide amount. It has previously been noted that as the amount of ceramide in the system increases, aggregates might develop, resulting in PS growth. One of the theories that have been anticipated to elucidate ceramide-brought structural changes was relied on the low capability of ceramide to pass through membranes. The addition of ceramide in the membranes might induce alternations in membrane curvature and subsequent PS increase (Castro et al., 2014). Regarding HA amount (X_2_), the PS of the formulated CCPCs increased by increasing HA amount, the previous findings might be related to the adsorption of HA in the wall of the vesicle establishing a coat that augmented the PS with rising in HA amount (Fahmy et al., 2021). The previous findings agreed with Tran et al., who found that augmenting HA concentration increased the PS markedly of vorinostat loaded solid lipid nanoparticles (SLNs) (Tran et al., 2014). For DDAB amount (X_3_), by increasing the DDAB amount the PS of the formulated vesicles increased (Berrin & Asuman, 2017). These findings were in accordance with Asasutjarit et al. as they prepared SLN and justified their results as follows: at the minor amount of DDAB, the PS of SLN decreased. This could be a result of lower interfacial tension through the oil phase and water phase by surface-active agents leading to an increment of the surface curvature of smaller oil droplets. However, when the DDAB amount was augmented the PS of SLN was increased which could be related to the deposition of extra surfactant at the SLN surface (Asasutjarit et al., 2007).

### Evaluation of PDI results

It’s widely recognized that PDI values near zero imply size homogeneity, whereas values near 1 indicate sample heterogeneity (Albash et al., 2021b). The PDI values (Table 3) ranged from 0.141 ± 0.06 to 0.591 ± 0.026. The PDI values of the prepared CCPCs justify their relative homogeneity. All factors were not significant *p* ≥ 0.05 subsequently PDI was removed from the optimization step.

### Effect of formulation variables on the ZP

**Figure 2.**
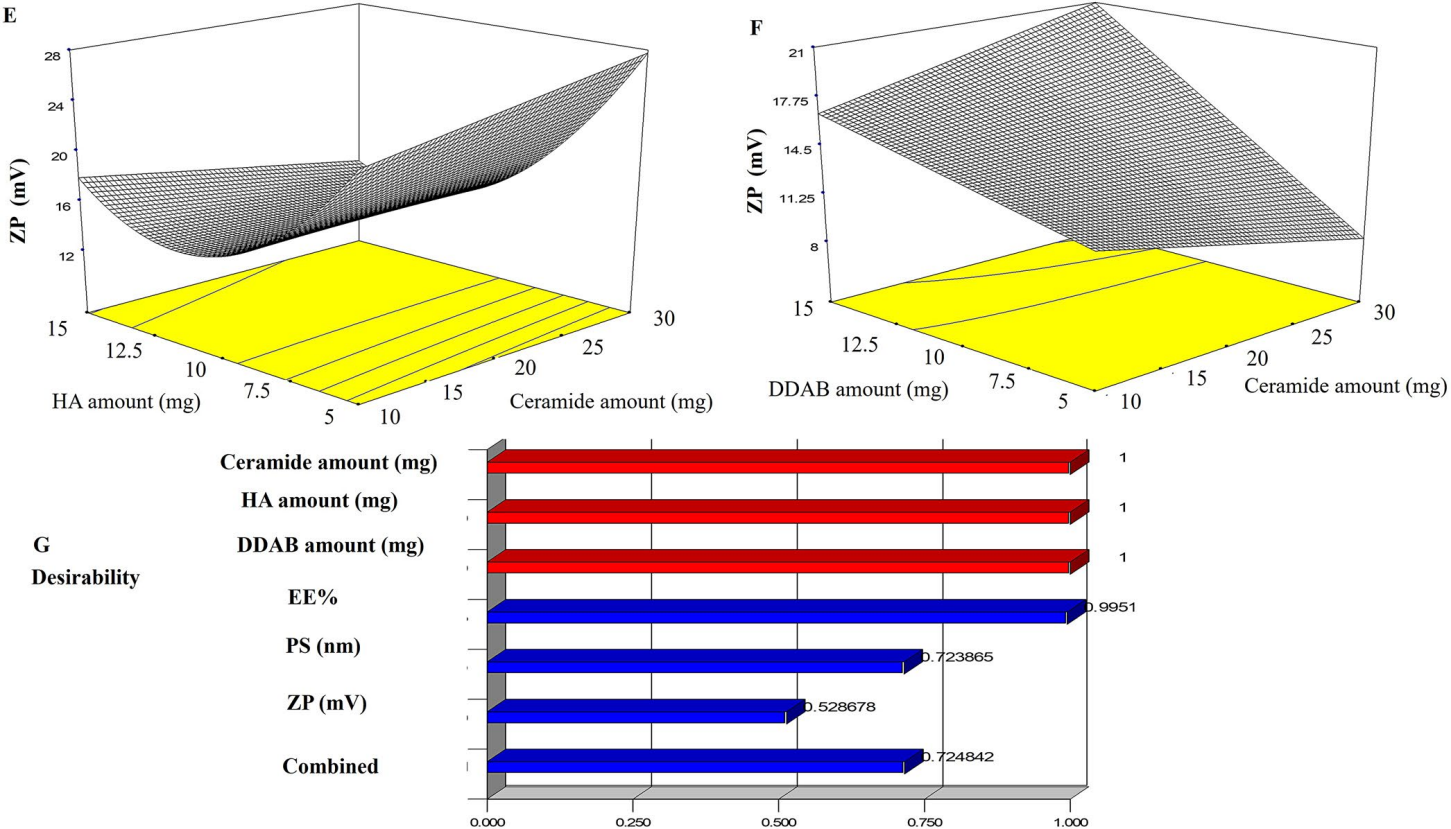
Effect of formulation variables on ZP of LVC-CCPCs (E-F) and desirability figure(G). Abbreviation: ZP: zeta potential; LVC: levocetirizine hydrochloride and CCPCs; cationic ceramide/phospholipid composite.

ZP of LVC-loaded CCPCs ranged from 7.56 ± 0.48 to 34.40 ± 1.55 mV (Table 3). The output data (Table 4), and Figure 2E–F depict the impact of the investigated variables on the ZP of the CCPCs. Ceramide amount (X_1_) showed no significant effect on ZP. On the other hand, HA amount (X_2_) and DDAB amount (X_3_) showed a significant effect with a p-value of < 0.0001 for both factors. For HA amount (X_2_), it was found that by increasing HA amount the positive ZP values of CCPCs decreased as HA possesses a negative surface charge that participates in decreasing ZP values (Tran et al., 2014). On the contrary, DDAB amount (X_3_) the results showed that the increase of the DDAB amount gradually augmented the ZP of CCPCs. This was correlated to DDAB molecules (cationic surfactant) could be present more at the interface of CCPCs hence, the positive ZP values increased (Asasutjarit et al., 2007).

### Optimization of CCPCs based on the desirability criterion

LVC-CCPCs were to be optimized via the constraints in Table 1 with the exclusion of the non-significant response (PDI). The optimum values of the variables were gained via numerical optimization relied on a desirability tool utilizing Design-Expert- 7^®^ software. A suggested CCPCs enclosing 10 mg ceramide, 5 mg HA, and 5 mg DDAB as a cationic surfactant (Figure 2G). The observed EE%, PS, and ZP results were 88.36 ± 0.34%, 479.00 ± 50.34 nm, and ZP of 20.20 ± 1.13 mV respectively. The predicted values were 88.84%, 484.53 nm, and 21.84 mV, respectively. The high similarity between the observed, and predicted values of the optimum CCPC could conclude the validity of the design to predict the responses (Table 4).

### Short term stability study

The values of fresh and stored optimum CCPCs are listed in Table 5. In terms of EE %, PS, ZP, and PDI, there was no statistical difference (*p* > 0.05 for all values). Furthermore, during the storage time, the physical appearance of optimum CCPCs did not change.

**Table 5.** Stability study of the optimum CCPCs.

Parameter*	Fresh CCPCs	Stored CCPCs (3 months)
**EE%**	88.36 ± 0.34	87.50 ± 1.60
**PS (nm)**	479.00 ± 50.34	467.80 ± 10.10
**PDI**	0.377 ± 0.003	0.356 ± 0.001
**ZP (mV)**	20.20 ± 1.13	19.50 ± 0.06

* Mean ± SD (*n* = 3).

Abbreviation: EE%: entrapment efficiency percent; PS: particle size; PDI: polydispersity index; ZP: zeta potential, and CCPCs; cationic ceramide phospholipid composite.

### Transmission electron microscopy (TEM)

**Figure 3.**
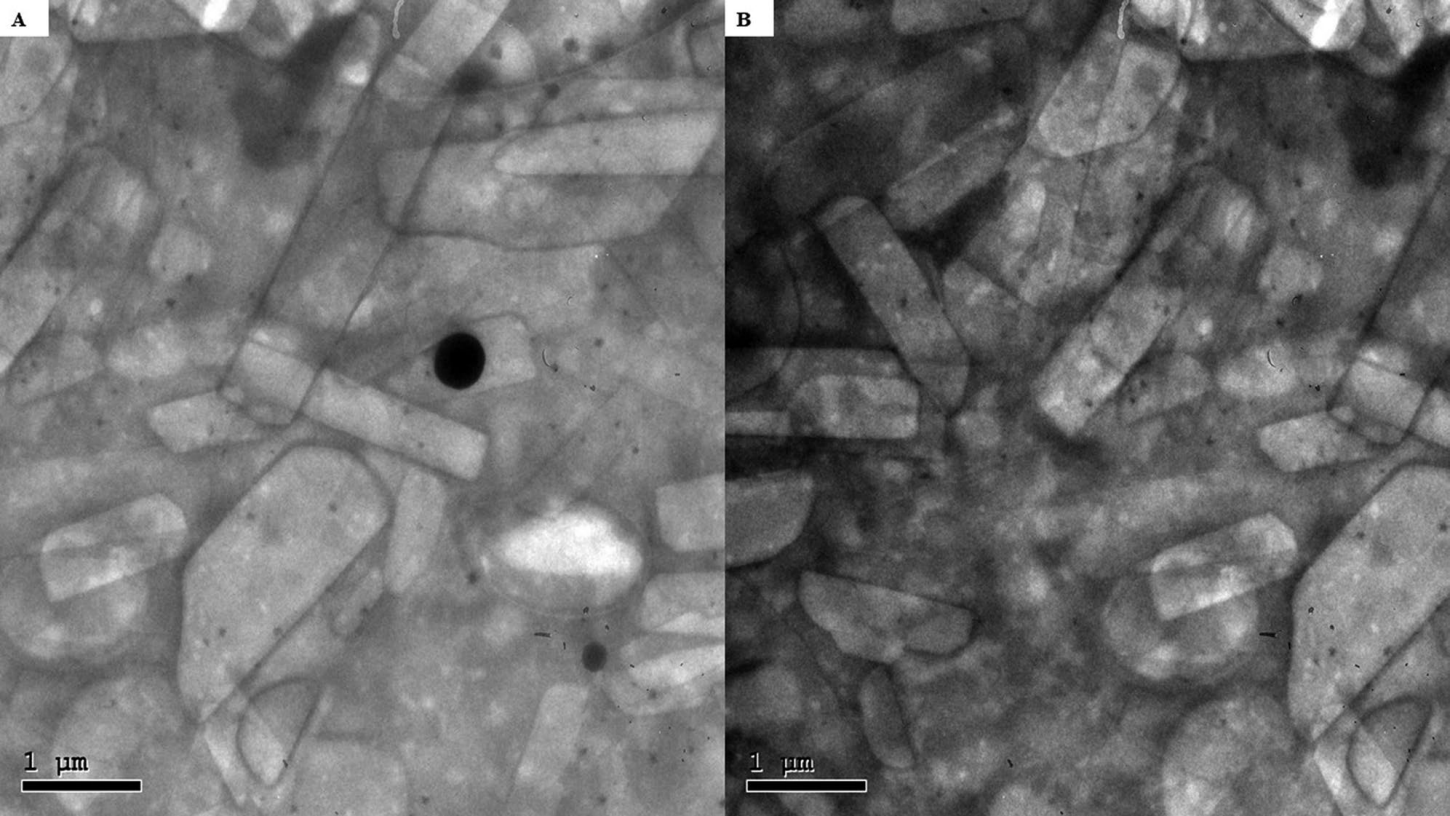
Transmission electron micrographs of LVC-loaded CCPCs. Abbreviation: LVC: levocetirizine hydrochloride and CCPCs; cationic ceramide/phospholipid composite.

When ceramide was incorporated into vesicles, TEM revealed a drastic alteration in the morphology of the vesicles. Additionally, as seen in Figure 3, the best CCPCs had fiber-like architecture with the production of elongated interwoven ceramide tubules. The micrographs revealed the existence of rounded vesicles in addition to tubular vesicles upon examination due to the presence of PC (Abdelgawad et al., 2017).

### *In-silico* studies

**Figure 4.**
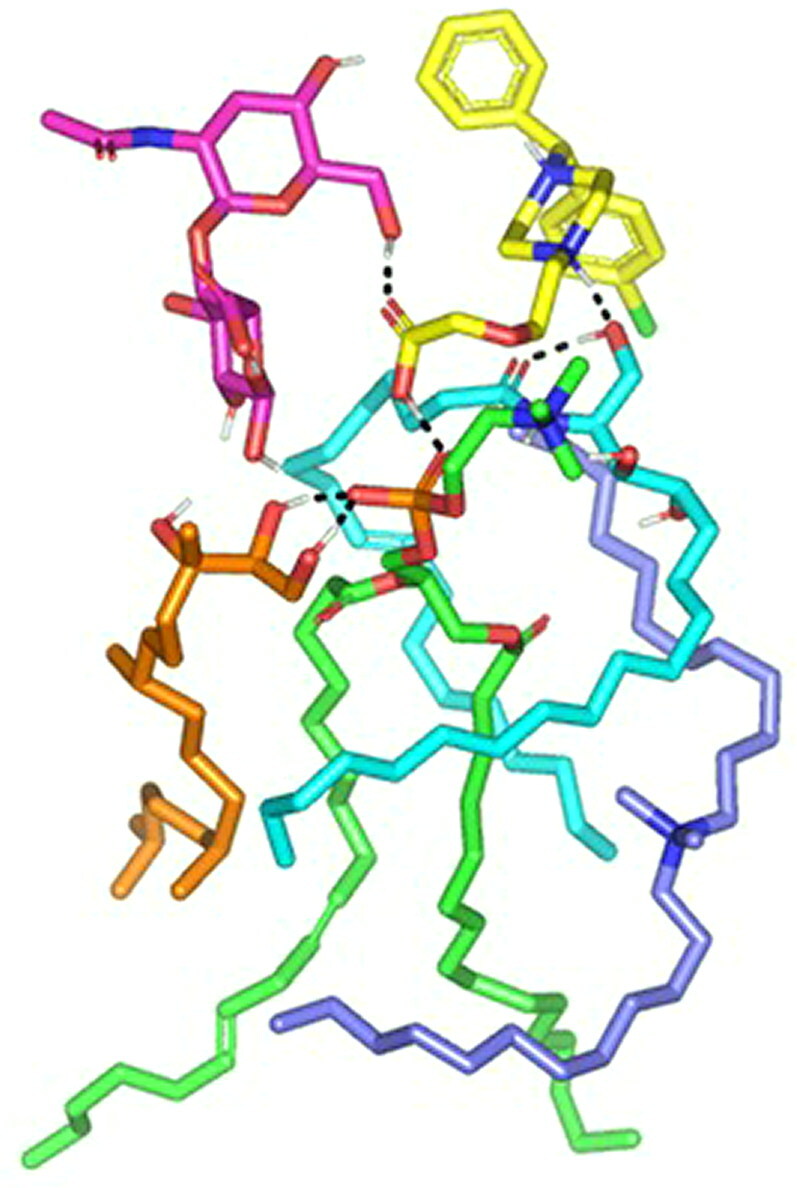
Predicted docking pose of LVC-PC binding complex. Stick 3 D-representation of LVC (yellow) loaded on PC interface (green), in presence of several CCPCs additives; ceramide (cyan), DDAB (blue), Phytanriol (orange), and HA (magenta). All introduced formulation additives served as either direct or indirect connecting/supportive platforms for favored anchoring of LVC within the drugphospholipid complex. Polar interactions (hydrogen bond) are represented as black dashed lines. Abbreviation: HA: hyaluronic acid; DDAB: dimethyldidodecylammonium bromide; PC: phospholipid; LVC: levocetirizine hydrochloride and CCPCs; cationic ceramide/phospholipid composite.

A molecular docking study was adopted for evaluating the magnitude and nature of binding interaction between LVC and PC in presence of CCPCs components; Ceramide, DDAB, phytantriol, and HA. Molecular docking investigation revealed the nature of binding among the investigated molecules where both polar/hydrogen bond and hydrophobic interactions have guided the ligand-target stability. Generally, LVC was anchored at the PC phosphate head exhibiting polar interaction with the PC’s polar phosphate head (–OPO(O)O^-^) through the ligand’s terminal carboxylic group (H-bond angle 163.10° length 1.20 Å) (Figure 4). The obtained LVC-PC pose was considered relevant since several reported small molecules, including the oral hypoglycemic agent metformin and hypolipidemic drug rosuvastatin and metformin, illustrated preferential orientations toward the phosphate scaffold of the PC molecule (Abd-Elsalam et al., 2018; Farag et al., 2021). Stability of the LVC-PC complex was further mediated through combined polar/hydrophobic interactions with CCPCs additives. Both ceramide and DDAB were settled around the PC acyl chains where dominant van der Waals energy potentials guided the anchoring of these earlier molecules at the PC’s hydrophobic scaffold. Despite the lack of polar contacts, the oxygen functionalities present within the ceramide structure were settled toward the phosphate polar head of PC for minimizing the potential electrostatic penalties associated with anchoring ceramide near the PC acyl tails. The latter ceramide/PC favored orientation was found beneficial for further stabilizing the LVC molecule at the PC interface where a strong close hydrogen bond was depicted between the ligands’ hydrogen bond donor and a ceramide hydroxyl group (H-bond angle 160.90° length 1.80 Å). It is worth noting that, the long hydrophobic aliphatic tails of ceramide allowed relevant van der Waal contacts with the LVC aromatic functionalities permitting additional stability for the LVC-PC complex. The electrostatic potentiality of LVC was also compensated through hydrogen bond interaction via the LVC’s carboxyl group with the highly polar nano-formulation additive, HA (H-bond angle 172.20° length 1.80 Å). Only phytantriol predicted no relevant direct interaction with the drug structure, however, this docked CCPCs additive exhibited double hydrogen bonding with the PC’s polar phosphate oxygen anion (H-bond angle 166.40° length 1.40 Å and angle 148.10° length 1.50 Å). The long branched aliphatic chain of phytantriol depicted extended orientation along with one of the PC’s acyl chains favoring highly close van der Waal contacts with both PC and the surrounding ceramide CCPCs molecules. Based on the above-described collaborative binding interactions among the CCPCs components, a high negative docking score was assigned for LVC in complex with the formulated PC molecule (−7.01 kCal/mol). These docking findings managed to explain the enhanced formulation parameters following the introduction of CCPCs additives where they managed to serve as carrier agents mediating LVC loading upon PC molecule for efficient drug solubilization.

**Figure 5.**
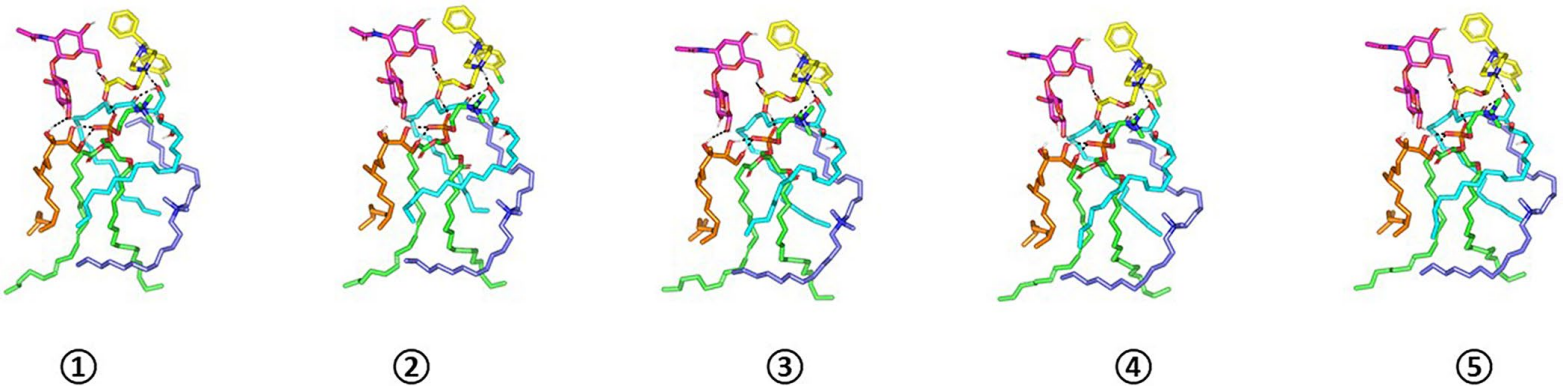
Time evolution conformational alterations of LVC-PC-CCPCs additive heterocomplex throughout an explicit MD simulation run at a 100% aqueous solvation system. The thermodynamic movements and stability of formulation components; LVC (yellow) loaded on PC interface (green), in presence of several CCPCs; Ceramide (cyan), DDAB (blue), Phytantriol (orange), and HA (magenta), were monitored over MD simulation trajectories being captured at different snapshots ① 0.2 ns, ② 0.4 ns; ③ 0.6 ns; ④ 0.8 ns; and ⑤ 1 ns. Polar interactions (hydrogen bond) are represented as black dashed lines. Abbreviation: HA: hyaluronic acid; DDAB: dimethyldidodecylammonium bromide; PC: phospholipid; LVC: levocetirizine hydrochloride and CCPCs; cationic ceramide/phospholipid composite; MD: molecular dynamic simulation.

For evaluating the thermodynamic stability of the docked LVC-PC complex, this model was subjected to explicit MD simulation within the formulation final solvent (100% aqueous solvation state). To our delight, the LVC-PC complex illustrated significant stability along with the whole MD simulation timeframe (1000 ps) (Figure 5). With a large negative free binding energy (−224.508 ± 23.44 Kcal/mol), the LVC illustrated preferential stability and affinity toward the PC carrier. Interestingly, Van der Waals hydrophobic potentials dominated the binding interaction energy contribution, whereas all initial polar contacts during the docking analysis were maintained along the whole MD simulation timeline. The LVC dynamic behavior was correlated with limited conformational and orientation drift (RMSD < 2.00 Å) following the beginning of the MD production stage.

On the other hand, both ceramide and DDAB showed more tight conformation/orientation toward the acyl chains of PC molecules favoring non-polar binding interactions. The extended hydrogen bond network between the polar functionality of LVC (COOH/piperazine *N*-atom) and the hydrophilic heads of both PC and ceramide molecules were maintained across the entire simulation run. A similar observation was illustrated for the HA-LVC hydrogen bond pair where the polar interaction between the drug’s carbonyl group and the HA’s hydroxylated sugar part was conserved along the MD simulation run. These latter hydrogen bond pairs illustrated optimal hydrogen bond distances and angles (1.28 Å up to 1.83 Å and 142.12° up to 163.52°) the thing that preserved the closeness through the formulation carrier additive/PC and the drug molecule.

**Figure 6.**
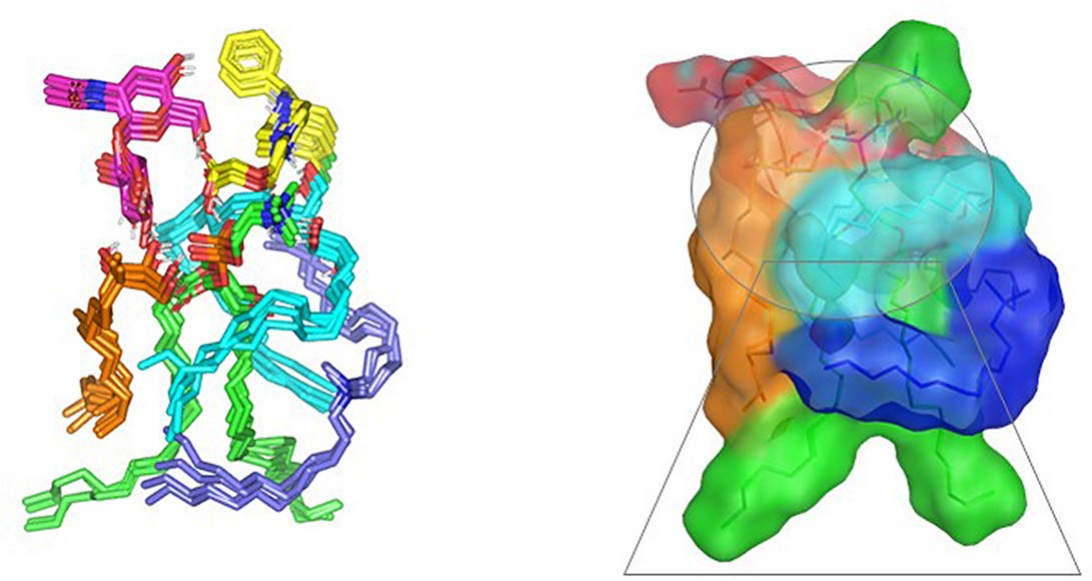
Overlay of LVC-PC-CCPCs additive heterocomplex across MD simulation frames (left panel) and molecular surface 3 D-representation of the inverted cone micellar configuration at 100% aqueous solvation system (right panel). Molecular surface and sticks 3 D representations were illustrated in colors being previously assigned for the optimized formulation components; yellow, green, cyan, blue, orange, and magenta for LVC, PC, Ceramide, DDAB, Phytantriol, and HA, respectively. Abbreviation: HA: hyaluronic acid; DDAB: dimethyldidodecylammonium bromide; PC: phospholipid; LVC: levocetirizine hydrochloride and CCPCs; cationic ceramide/phospholipid composite; MD: molecular dynamic simulation.

Concerning the stability of phytantriol across the MD simulation run, the initial double polar binding interactions with the PC carrier were also conserved along the simulated trajectories (1.46 ± 0.05 Å/167.00 ± 4.27° and 1.34 ± 0.04 Å/166.58 ± 5.13°). Notably, phytantriol was further stabilized at the PC interface through an additional transient hydrogen bonding with the C_1_-OH group of HA glycosidic moiety around 0.2 ns and 0.6 ns of the MD simulation run (2.65 ± 0.07 Å/138.8 ± 10.89°). In additional to polar-mediated HA stability, the depicted stable aliphatic chain orientation of phytantriol across the simulation timeframe would further highlight the significant role of phytantriol-PC hydrophobic contacts in stabilizing this aliphatic formulation additive at the LVC-PC complex interface. Finally, the spatial conformation of PC lipophilic elongated chains showed interesting findings. The conserved polar contacts at the phosphate group of the PC carrier permitted both the PC hydrophobic acyl tails to be pulled away from each other. The later dynamic behavior furnished an open-compass confirmation for the PC extended tails the thing that would have increased the volume of the hydrophobic chain. On the contrary, a small surface area was preserved along the simulation run since the PC-complex established various strong compact hydrogen bond interactions at the phosphate polar head. Such type of packing made the drug-PC complex attain an inverted cone shape with maintained micellar conformation being previously reported with several small molecules (Figure 6).

### *Ex-vivo* studies

**Figure 7.**
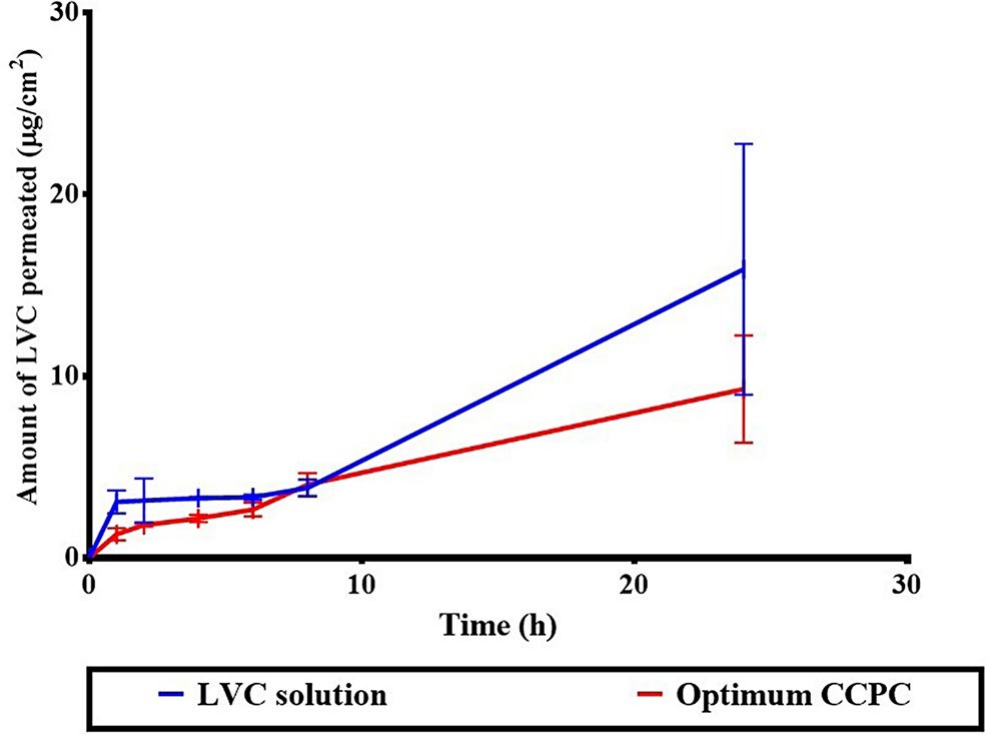
*Ex-vivo* permeation profile of LVC from CCPCs, compared to its aqueous solution. Abbreviation: LVC: levocetirizine hydrochloride and CCPCs; cationic ceramide/phospholipid composite.

Encapsulation of LVC within CCPCs has resulted in its retention, reducing its permeation in comparison to its solution (Figure 7). This was demonstrated by much lower Q24 (total amount penetrated per unit area after 24 hours) and J_max_ values, as well as a significantly higher Dep24 value, resulting in an LAEI value 2.05 times higher than the LVC solution (*p* < 0.05) (Table 6). Formulation constituents, HA and ceramide III are described to augment the localization of drugs, while decreasing their penetration into the blood. HA is known as a high molecular weight and hydrophilic molecule, hindering the permeation of drugs and might aid their successful retention (Kasetvatin et al., 2015). Additionally, we speculate that HA is not acting as an enhancer for hydrophilic drugs hence it more likely acting as a carrier that aided in sufficient LVC encapsulation inside CCPCs and in addition augmented its deposition thorough skin layer. In addition, Abdelgawad et al. reported that ceramide-containing vesicles (cerosomes) enhanced the retention of tazarotene through skin layers due to the presence of ceramide (Abdelgawad et al., 2017).

**Table 6.** Permeation of LVC solution compared to the optimum CCPCs and permeation parameters.

Time (hr)	LVC solution (μg/ml)	CCPC (μg/ml)
**1**	3.07 ± 0.63	1.29 ± 0.33
**2**	3.15 ± 1.21	1.80 ± 0.09
**4**	3.28 ± 0.06	2.16 ± 0.20
**6**	3.33 ± 0.15	2.65 ± 0.38
**8**	3.84 ± 0.46	4.02 ± 0.64
**24**	15.87 ± 6.90	9.28 ± 2.96
**Permeation parameters**	**LVC solution (μg/ml)**	**CCPC (μg/ml)**
**Q_24_ (mg/cm^2^)**	15.87 ± 6.90	9.28 ± 2.96
**Dep_24_ (mg/cm^2^)**	146.47 ± 27.64	202.38 ± 14.45
**J_max_ (mg/h cm^2^)**	1.32 ± 0.57	0.77 ± 0.24
**LAEI**	12.04 ± 6.47	24.69 ± 8.99
**LAEI ratio**		2.05

* Mean ± SD (*n* = 3).

Q_24_: cumulative amount permeated per unit area after 24 h, Dep_24_: cumulative amount deposited per unit area after 24 h, *J*_max_: maximum average flux after 24 h, LAEI: Local accumulation efficiency index; LVC: levocetirizine hydrochloride, and CCPCs: cationic ceramide/phospholipid composite.

### *In-vivo* studies

#### *In-vivo* dermatokinetic assessment

**Figure 8.**
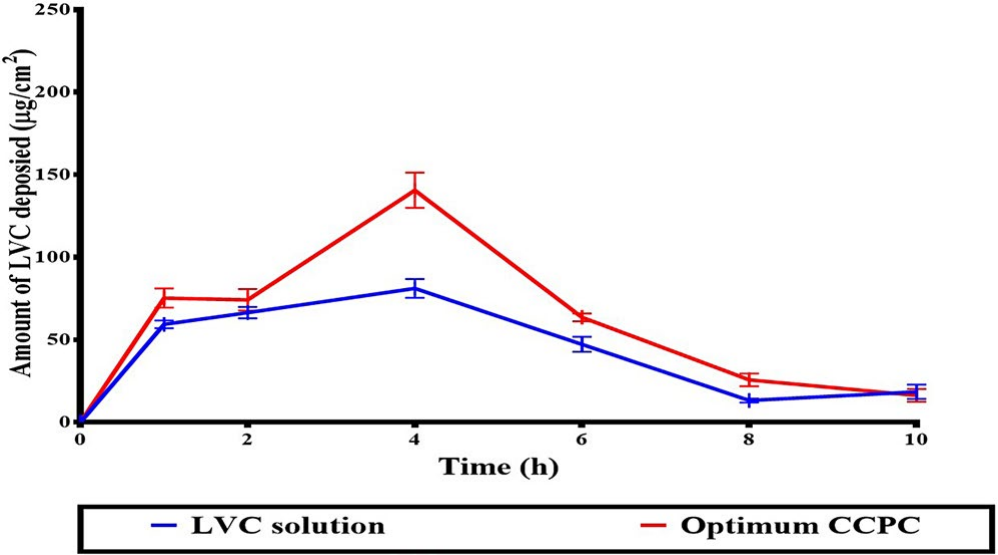
*In-vivo* skin deposition profile of LVC from CCPCs, compared to its aqueous solution. Abbreviation: LVC: levocetirizine hydrochloride, and CCPCs; cationic ceramide/phospholipid composite.

The deposition profile of LVC from the optimum CCPCs, related to the LVC solution is depicted in Figure 8. LVC was retained from CCPCs in higher amounts related to that from the solution. The AUC_0–10_, obtained from the deposition profiles was 706.66 ± 20.45 µg.h/cm^2^ for CCPCs, which was significantly higher than that of the LVC solution (553.58 ± 30.87 µg.h/cm^2^), meaning a 1.2 fold increase in LVC-deposition. These results support the findings of the *ex-vivo* investigation, indicating that both HA and ceramide have an effect on the localization of LVC in the skin. The skin treated with CCPCs showed significantly (*p* < 0.05) higher C_max_ of 140.61 ± 9.76 compared to LVC solution of 81.06 ± 8.99 µg/ml. Furthermore, the T_max_ of both LVC solution and optimum CCPCs was 4 hour.

#### Histopathologic evaluation

**Figure 9.**
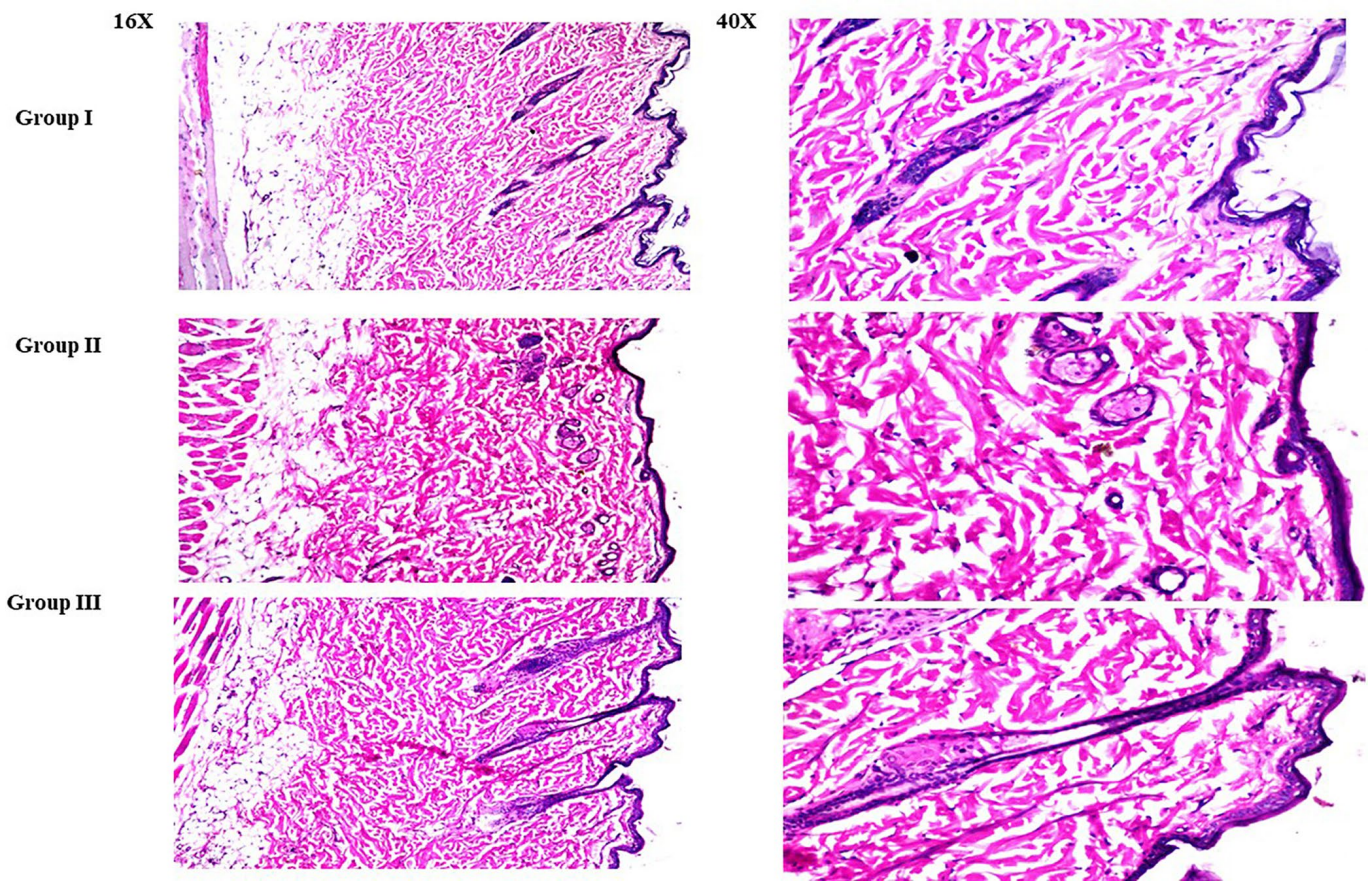
Light microscope photomicrographs showing histopathological sections (hematoxylin and eosin-stained) of rat skin in normal control (group I), rat skin treated with LVC solution (group II), and rat skin treated with optimum CCPCs (group III) with a magnification power of 16X to illustrate all skin layers (Left side) and magnification power of 40X to identify the epidermis and dermis (Right side). Abbreviation: LVC: levocetirizine hydrochloride, and CCPCs; cationic ceramide/phospholipid composite.

In comparison to untreated skin sections, rats treated with LVC solution (group II) and CCPCs (group III) did not demonstrate any histological alterations in their skin (group I) (Figure 9). These findings verified the safety of CCPCs for topical application for alopecia management.

## Conclusion

Levocetirizine hydrochloride (LVC) due to its capability to inhibit prostaglandin expression might enhance hair proliferation. Such impacts are independent of LVC antihistaminic action. LVC loaded cationic ceramide/phospholipid composite (CCPCs) were fabricated by ethanol injection, via central composite design. The optimum CCPCs were stable for 90 days. Transmission electron microscopy viewed the tubular vesicular shape of the optimum formula. *The in-silico* study verified the capability of formulation components to bind effectively and produce a stable formulation. Both *ex-vivo* and *in-vivo* dermatokinetic assessments showed better deposition of LVC from the optimum formula, related to its solution. Histopathology verified the safety of the optimum formula. Subsequently, it could be considered that the optimum formula might be an effective carrier that could manage alopecia effectively.
